# Publisher Correction: Computer-aided interpretation of chest radiography reveals the spectrum of tuberculosis in rural South Africa

**DOI:** 10.1038/s41746-021-00485-6

**Published:** 2021-07-16

**Authors:** Jana Fehr, Stefan Konigorski, Stephen Olivier, Resign Gunda, Ashmika Surujdeen, Dickman Gareta, Theresa Smit, Kathy Baisley, Sashen Moodley, Yumna Moosa, Willem Hanekom, Olivier Koole, Thumbi Ndung’u, Deenan Pillay, Alison D. Grant, Mark J. Siedner, Christoph Lippert, Emily B. Wong, the Vukuzazi Team, Anand Ramnanan, Anele Mkhwanazi, Antony Rapulana, Anupa Singh, Ashentha Govender, Ayanda Zungu, Boitsholo Mfolo, Bongani Magwaza, Bongumenzi Ndlovu, Clive Mavimbela, Costa Criticos, Day Munatsi, Dilip Kalyan, Doctar Mlambo, Fezeka Mfeka, Freddy Mabetlela, Gregory Ording-Jespersen, Hannah Keal, Hlengiwe Dlamini, Hlengiwe Khathi, Hlobisile Chonco, Hlobisile Gumede, Hlolisile Khumalo, Hloniphile Ngubane, Hollis Shen, Hosea Kambonde, Innocentia Mpofana, Jabu Kwinda, Jaco Dreyer, Jade Cousins, Jaikrishna Kalideen, Janet Seeley, Kandaseelan Chetty, Kayleen Brien, Kennedy Nyamande, Kgaugelo Moropane, Khabonina Malomane, Khadija Khan, Khanyisani Buthelezi, Kimeshree Perumal, Kobus Herbst, Lindani Mthembu, Logan Pillay, Mandisi Dlamini, Mandlakayise Zikhali, Mbali Mbuyisa, Mbuti Mofokeng, Melusi Sibiya, Mlungisi Dube, Mosa Suleman, Mpumelelo Steto, Mzamo Buthelezi, Nagavelli Padayachi, Nceba Gqaleni, Ngcebo Mhlongo, Nokukhanya Ntshakala, Nomathamsanqa Majozi, Nombuyiselo Zondi, Nomfundo Luthuli, Nomfundo Ngema, Nompilo Buthelezi, Nonceba Mfeka, Nondumiso Khuluse, Nondumiso Mabaso, Nondumiso Zitha, Nonhlanhla Mfekayi, Nonhlanhla Mzimela, Nozipho Mbonambi, Ntombiyenhlanhla Mkhwanazi, Ntombiyenkosi Ntombela, Pamela Ramkalawon, Pfarelo Tshivase, Phakamani Mkhwanazi, Philippa Mathews, Phumelele Mthethwa, Phumla Ngcobo, Ramesh Jackpersad, Raynold Zondo, Rochelle Singh, Rose Myeni, Sanah Bucibo, Sandile Mthembu, Sashin Harilall, Senamile Makhari, Seneme Mchunu, Senzeni Mkhwanazi, Sibahle Gumbi, Siboniso Nene, Sibusiso Mhlongo, Sibusiso Mkhwanazi, Sibusiso Nsibande, Simphiwe Ntshangase, Siphephelo Dlamini, Sithembile Ngcobo, Siyabonga Nsibande, Siyabonga Nxumalo, Sizwe Ndlela, Skhumbuzo Mthombeni, Smangaliso Zulu, Sphiwe Clement Mthembu, Sphiwe Ntuli, Talente Ntimbane, Thabile Zondi, Thandeka Khoza, Thengokwakhe Nkosi, Thokozani Bhengu, Thokozani Simelane, Tshwaraganang Modise, Tumi Madolo, Velile Vellem, Welcome Petros Mthembu, Xolani Mkhize, Zamashandu Mbatha, Zinhle Buthelezi, Zinhle Mthembu, Zizile Sikhosana

**Affiliations:** 1 Africa Health Research Institute, KwaZulu-Natal, South Africa; 2 Digital Health & Machine Learning, Hasso Plattner Institute for Digital Engineering, Berlin, Germany; 3 Hasso Plattner Institute for Digital Health at Mount Sinai, Icahn School of Medicine at Mount Sinai, New York, NY USA; 4 School of Nursing and Public Health, College of Health Sciences, University of KwaZulu-Natal, Durban, South Africa; 5 Division of Infection and Immunity, University College London, London, UK; 6 London School of Hygiene & Tropical Medicine, London, UK; 7 HIV Pathogenesis Programme, The Doris Duke Medical Research Institute, University of KwaZulu-Natal, Durban, South Africa; 8 Ragon Institute of MGH, MIT and Harvard University, Cambridge, MA USA; 9 Max Planck Institute for Infection Biology, Berlin, Germany; 10 School of Clinical Medicine, College of Health Sciences, University of KwaZulu-Natal, Durban, South Africa; 11 Harvard Medical School, Boston, MA USA; 12 Division of Infectious Diseases, Massachusetts General Hospital, Boston, MA USA; 13 Division of Infectious Diseases, University of Alabama at Birmingham, Birmingham, AL USA; 14 Aurum Innova (Pty) Ltd, Parktown, Johannesburg, South Africa; 15 iMarketing Consultants, Windhoek West, Windhoek, Namibia; 16 Perumal and Partners, Mayville, Durban, South Africa; 17 Department of Pulmonology, Nelson R. Mandela School of Medicine, University of KwaZulu-Natal, Durban, South Africa; 18 Traditional Medicine Laboratory, School of Nursing & Public Health, College of Health Sciences, University of KwaZulu-Natal, Durban, South Africa; 19 Jacpersad Inc, Overport, Durban, South Africa

**Keywords:** Tuberculosis, Epidemiology

Correction to: *npj Digital Medicine* 10.1038/s41746-021-00471-y, published online 2 July 2021

The original version of this Article contained errors in the affiliations of members of the Vukuzazi Team.

Anand Ramnanan, Anele Mkhwanazi, Antony Rapulana, Anupa Singh, Ashentha Govender, Ayanda Zungu, Bongani Magwaza, Bongumenzi Ndlovu, Clive Mavimbela, Costa Criticos, Day Munatsi, Dilip Kalyan, Doctar Mlambo, Fezeka Mfeka, Freddy Mabetlela, Gregory Ording-Jespersen, Hannah Keal, Hlengiwe Dlamini, Hlengiwe Khathi, Hlobisile Chonco, Hlobisile Gumede, Hlolisile Khumalo, Hloniphile Ngubane, Hollis Shen, Innocentia Mpofana, Jaco Dreyer, Jade Cousins, Kandaseelan Chetty, Kayleen Brien, Khadija Khan, Khanyisani Buthelezi, Kimeshree Perumal, Kobus Herbst, Lindani Mthembu, Logan Pillay, Mandisi Dlamini, Mandlakayise Zikhali, Mbali Mbuyisa, Mbuti Mofokeng, Melusi Sibiya, Mlungisi Dube, Mpumelelo Steto, Mzamo Buthelezi, Nagavelli Padayachi, Nceba Gqaleni, Ngcebo Mhlongo, Nokukhanya Ntshakala, Nomathamsanqa Majozi, Nombuyiselo Zondi, Nomfundo Luthuli, Nomfundo Ngema, Nompilo Buthelezi, Nonceba Mfeka, Nondumiso Khuluse, Nondumiso Mabaso, Nondumiso Zitha, Nonhlanhla Mfekayi, Nonhlanhla Mzimela, Nozipho Mbonambi, Ntombiyenhlanhla Mkhwanazi, Ntombiyenkosi Ntombela, Pamela Ramkalawon, Phakamani Mkhwanazi, Philippa Mathews, Phumelele Mthethwa, Phumla Ngcobo, Raynold Zondo, Rochelle Singh, Rose Myeni, Sanah Bucibo, Sandile Mthembu, Sashin Harilall, Senamile Makhari, Seneme Mchunu, Senzeni Mkhwanazi, Sibahle Gumbi, Siboniso Nene, Sibusiso Mhlongo, Sibusiso Mkhwanazi, Sibusiso Nsibande, Simphiwe Ntshangase, Siphephelo Dlamini, Sithembile Ngcobo, Siyabonga Nsibande, Siyabonga Nxumalo, Sizwe Ndlela, Skhumbuzo Mthombeni, Smangaliso Zulu, Sphiwe Clement Mthembu, Sphiwe Ntuli, Talente Ntimbane, Thabile Zondi, Thandeka Khoza, Thengokwakhe Nkosi, Thokozani Bhengu, Thokozani Simelane, Tshwaraganang Modise, Tumi Madolo, Welcome Petros Mthembu, Xolani Mkhize, Zamashandu Mbatha, Zinhle Buthelezi, Zinhle Mthembu and Zizile Sikhosana were incorrectly associated with “Digital Health & Machine Learning, Hasso Plattner Institute for Digital Engineering, Berlin, Germany” and the affiliation “Africa Health Research Institute, KwaZulu-Natal, South Africa” was inadvertently omitted.

This has now been corrected in both the PDF and HTML versions of the Article.

