# Supplementary material for: Computer-aided interpretation of chest radiography reveals the spectrum of tuberculosis in rural South Africa
Source: NPJ Digit Med. 2021 Jul 2;4:106. doi: 10.1038/s41746-021-00471-y (PMC8253848; doi:10.1038/s41746-021-00471-y)
Supplement: Supplementary file 1 — Reporting Summary [file 41746_2021_471_MOESM1_ESM.pdf]

nature research

Corresponding author(s): Dr. Emily Wong

Last updated by author(s): 29/4/2021

Reporting Summary

Nature Research wishes to improve the reproducibility of the work that we publish. This form provides structure for consistency and transparency in reporting. For further information on Nature Research policies, see our Editorial Policies and the Editorial Policy Checklist.

Statistics

For all statistical analyses, confirm that the following items are present in the figure legend, table legend, main text, or Methods section.

n/a	Confirmed	
☐	☒	The exact sample size (n) for each experimental group/condition, given as a discrete number and unit of measurement
☐	☒	A statement on whether measurements were taken from distinct samples or whether the same sample was measured repeatedly
☐	☒	The statistical test(s) used AND whether they are one- or two-sided<br>*Only common tests should be described solely by name; describe more complex techniques in the Methods section.*
☐	☒	A description of all covariates tested
☐	☒	A description of any assumptions or corrections, such as tests of normality and adjustment for multiple comparisons
☐	☒	A full description of the statistical parameters including central tendency (e.g. means) or other basic estimates (e.g. regression coefficient) AND variation (e.g. standard deviation) or associated estimates of uncertainty (e.g. confidence intervals)
☐	☒	For null hypothesis testing, the test statistic (e.g. F, t, r) with confidence intervals, effect sizes, degrees of freedom and P value noted<br>*Give P values as exact values whenever suitable.*
☐	☒	For Bayesian analysis, information on the choice of priors and Markov chain Monte Carlo settings
☐	☒	For hierarchical and complex designs, identification of the appropriate level for tests and full reporting of outcomes
☐	☒	Estimates of effect sizes (e.g. Cohen's d, Pearson's r), indicating how they were calculated

Our web collection on statistics for biologists contains articles on many of the points above.

Software and code

Policy information about availability of computer code

Data collection

The multi-disease community-based screening program ‘Vukuzazi’ used mobile vans to provide free health assessments in the rural uMkhanyakude district of KwaZulu-Natal in South Africa. The data for this analysis was collected during the first year of the project (between 25 May 2018 and 24 May 2019).36 Study field workers visited households to explain and provide the study description and invite eligible residents to participate. Eligibility criteria included a minimum age of 15 years and ongoing residency in the area. Participants provided written informed consent to participate in the study. In the case of participant age under 18, consent was also obtained from the parents or guardians. At the camp, participants answered questions in their preferred language about smoking, TB symptoms and history, HIV history and treatment, and the information was captured digitally.35 HIV status was assessed by 4th generation antibody/antigen test (Genscreen Ultra HIV Ag-Ab, Bio-Rad, Marnes-la-Coquette, France) on venous blood.
Posterior-anterior digital CXRs were obtained by a health worker using a mobile unit (Canon CXDI-NE) and the obtained digital CXRs were uploaded uncompressed in DICOM-format into a cloud storage. On a local workstation at the camp, the commercially-available software CAD4TBv5 calculated scores off-line for each CXR, indicating lung abnormalities and likelihood of active pulmonary tuberculosis13,14,37. CAD4TB’s methodology is based on initial lung field segmentation and subsequent analysis of the lung shape, symmetry, and costophrenic angles, resulting in an abnormality score between 0 and 100 (increasing with abnormality).38 The output score reflects the probability of active TB visible on the CXR.14 Following WHO guidelines for TB-prevalence surveys10, participants were referred for sputum examination if they reported any cardinal TB-symptom (fever, weight loss, cough, or night sweats) or if they had an abnormal CXR (indicated by a CAD4TBv5 score equals or higher than the triaging threshold in the camp). CAD4TBv6, an updated version of the image interpretation software that uses deep neural networks,14 became available after data collection. CAD4TBv6 scores were calculated and analyzed retrospectively. Sputum specimens were analyzed for Mtb using Xpert Ultra MTB/RIF® (XpertUltra) (Cepheid, Sunnyvale, CA, USA) and liquid BACTEC MGIT culture (MGIT) (Becton Dickinson, UK), held for 42 days. Within seven days of enrolment, an expert radiologist with more than 35 years of local experience reviewed all CXRs from high-resolution DICOM files on a computer screen in a central setting, blinded to CAD4TBv5 scores and any patient information. The radiologist categorized each CXR as 1) having either normal or abnormal lung fields and if abnormal 2) as having radiological signs diagnostic of active TB.

Data analysis	We compared the categorization of any lung field abnormality by the radiologist and CAD4TBv5 and v6 scores to diagnostic definitions of TB (definite TB, definite TB trace excluded and probable TB). Performance was assessed by sensitivity, specificity, negative predictive value (NPV), precision (PPV), Area under the receiver operating curve (AUC) and area under precision-recall curve (PRAUC). Estimations of 95% confidence intervals (CI) are given. AUCs between CAD4TBv5 and v6 and HIV-positive and negative were compared using the DeLong method. Similar to Qin et al.,19 we calculated the number of needed sputum tests (NNT) and the number of missed TB cases for the radiologist and each CAD4TB score from 0-100. We defined NNT as the number of participants with a CAD4TB score equal or higher than the threshold value. Missed TB cases were defined as the number of TB cases below each threshold. Missed TB cases were obtained for definite TB, definite TB trace excluded and probable TB definitions and stratified by symptom status. We compared CAD4TB scores between diagnostic groups of definite and probable TB, stratified by HIV status and assessed significant differences using two-sided Mann-Whitney-Wilcoxon tests.<br>Data analysis was performed with R (version 4.0.3) using the packages 'epiR', 'pROC', and 'precrec'.

For manuscripts utilizing custom algorithms or software that are central to the research but not yet described in published literature, software must be made available to editors and reviewers. We strongly encourage code deposition in a community repository (e.g. GitHub). See the Nature Research guidelines for submitting code & software for further information.

Data

Policy information about availability of data

All manuscripts must include a data availability statement. This statement should provide the following information, where applicable:

- Accession codes, unique identifiers, or web links for publicly available datasets
- A list of figures that have associated raw data
- A description of any restrictions on data availability

The Vukuzazi screening protocol and anonymized data is available at https://data.ahri.org/index.php/catalog/990

Field-specific reporting

Please select the one below that is the best fit for your research. If you are not sure, read the appropriate sections before making your selection.

☒ Life sciences ☐ Behavioural & social sciences ☐ Ecological, evolutionary & environmental sciences

For a reference copy of the document with all sections, see nature.com/documents/nr-reporting-summary-flat.pdf

Life sciences study design

All studies must disclose on these points even when the disclosure is negative.

Sample size	n=9914
Data exclusions	n=406. 406 participants could not have chest radiography due to pregnancy or physical inability to climb into the mobile van;
Replication	not applicable
Randomization	not applicable
Blinding	The radiologist who was blinded to CAD4TB scores and all patient data such as age, history of TB, microbiological test results etc, interpreted each CXR in a central reading setting and assessed whether a) lung fields were normal or abnormal, and b) if abnormal, whether the findings were diagnostic of active TB.

Reporting for specific materials, systems and methods

We require information from authors about some types of materials, experimental systems and methods used in many studies. Here, indicate whether each material, system or method listed is relevant to your study. If you are not sure if a list item applies to your research, read the appropriate section before selecting a response.

Materials & experimental systems

n/a	Involved in the study	
☒	☐	Antibodies
☒	☐	Eukaryotic cell lines
☒	☐	Palaeontology and archaeology
☒	☐	Animals and other organisms
☐	☒	Human research participants
☒	☐	Clinical data
☒	☐	Dual use research of concern

Methods

n/a	Involved in the study	
☒	☐	ChIP-seq
☒	☐	Flow cytometry
☒	☐	MRI-based neuroimaging

Human research participants

Policy information about studies involving human research participants

Population characteristics	The median age of participants in the survey was 39 (interquartile range (IQR) 24-59), 6,664 (67.4%) were female, 29.8% (2,954 of 9,914) were HIV-positive and 10.7% (1,056 of 9,914) presented with at least one TB related symptom. 1,196 (12.1) reported to have been treated for TB in the past. 726 (7.3%) reported to be smokers.
Recruitment	The multi-disease community-based screening program 'Vukuzazi' used mobile vans to provide free health assessments in the rural uMkhanyakude district of KwaZulu-Natal in South Africa. The data for this analysis was collected during the first year of the project (between 25 May 2018 and 24 May 2019).36 Study field workers visited households to explain and provide the study description and invite eligible residents to participate. Eligibility criteria included a minimum age of 15 years and ongoing residency in the area. Participants provided written informed consent to participate in the study. In the case of participant age under 18, consent was also obtained from the parents or guardians. Approximately half of the eligible population enrolled in Vukuzazi, the multi-disease screening protocol. Reasons for participation and non-participation are discussed in Ngwenya et al, 2020 (Reference #36 in manuscript).
Ethics oversight	Ethics approval was obtained from the University of KwaZulu-Natal Biomedical Research Ethics Committee (BE560/17), the London School of Hygiene & Tropical Medicine Ethics Committee (14722), and the Partners Institutional Review Board (2018P001802).

Note that full information on the approval of the study protocol must also be provided in the manuscript.
