# Supplementary material for: Computer-aided interpretation of chest radiography reveals the spectrum of tuberculosis in rural South Africa
Source: NPJ Digit Med. 2021 Jul 2;4:106. doi: 10.1038/s41746-021-00471-y (PMC8253848; doi:10.1038/s41746-021-00471-y)
Supplement: Supplementary file 2 — Supplementary Information [file 41746_2021_471_MOESM2_ESM.pdf]

## Supplementary Information

Computer-aided interpretation of chest radiography reveals the spectrum of tuberculosis in rural South Africa

Jana Fehr[1,2], Stefan Konigorski[2,3], Stephen Olivier[1], Resign Gunda[1,4,5], Ashmika Surujdeen[1], Dickman Gareta[1], Theresa Smit[1], Kathy Baisley[1,6], Sashen Moodley[1], Yumna Moosa[1], Willem Hanekom[1,5], Olivier Koole[1,6], Thumbi Ndung'u[1,5,7,8,9], Deenan Pillay[1,5], Alison D. Grant[1,4,6,10], Mark J. Siedner[1,10,11,12], Christoph Lippert*[2,3], Emily B. Wong*[1,11,12,13] and the Vukuzazi Team[1,4-19]†.
* These authors contributed equally

†Vukuzazi-Team:

* Denotes team members who were closely involved with the design, implementation and oversight of Vukuzazi.

*Alison D. Grant[1,4,6,10], Anand Ramnanan[1], Anele Mkhwanazi[1], Antony Rapulana[1], Anupa Singh[1], Ashentha Govender[1], Ashmika Surujdeen[1], Ayanda Zungu[1], Boitsholo Mfolo[14], Bongani Magwaza[1], Bongumenzi Ndlovu[1], Clive Mavimbela[1], Costa Criticos[1], *Day Munatsi[1], *Deenan Pillay[1,5], *Dickman Gareta[1], Dilip Kalyan[1], Doctar Mlambo[1], *Emily B. Wong[1,11,11,13], Fezeka Mfeka[1], Freddy Mabetlela[1], *Gregory Ording-Jespersen[1], Hannah Keal[1], Hlengiwe Dlamini[1], Hlengiwe Khathi[1], Hlobisile Chonco[1], Hlobisile Gumede[1], Hlolisile Khumalo[1], Hloniphile Ngubane[1,] *Hollis Shen[1], Hosea Kambonde[15], *Innocentia Mpofana[1], Jabu Kwinda[14], *Jaco Dreyer[1], Jade Cousins[1], Jaikrishna Kalideen[16], *Janet Seeley[6], Kandaseelan Chetty[1], Kayleen Brien[1], Kennedy Nyamande[17], Kgaugelo Moropane[14], Khabonina Malomane[14], *Kathy Baisley[1,6], *Khadija Khan[1], Khanyisani Buthelezi[1], Kimeshree Perumal[1], *Kobus Herbst[1], Lindani Mthembu[1], Logan Pillay[1], Mandisi Dlamini[1], Mandlakayise Zikhali[1], *Mark J. Siedner[1,10,11,12], Mbali Mbuyisa[1], Mbuti Mofokeng[1], Melusi Sibiya[1], Mlungisi Dube[1], Mosa Suleman[17], Mpumelelo Steto[1], Mzamo Buthelezi[1], Nagavelli Padayachi[1], Nceba Gqaleni[1,18], *Ngcebo Mhlongo[1], Nokukhanya Ntshakala[1], Nomathamsanqa Majozi[1], Nombuyiselo Zondi[1], Nomfundo Luthuli[1], Nomfundo Ngema[1], Nompilo Buthelezi[1], Nonceba Mfeka[1], Nondumiso Khuluse[1], Nondumiso Mabaso[1], Nondumiso Zitha[1], Nonhlanhla Mfekayi[1], Nonhlanhla Mzimela[1], Nozipho Mbonambi[1], Ntombiyenhlanhla Mkhwanazi[1], Ntombiyenkosi Ntombela[1], *Olivier Koole[1,6], Pamela Ramkalawon[1], Pfarelo Tshivase[17], Phakamani Mkhwanazi[1], Philippa Mathews[1], Phumelele Mthethwa[1], Phumla Ngcobo[1], Ramesh Jackpersad[19], Raynold Zondo[1], *Resign Gunda[1,4,5], Rochelle Singh[1], Rose Myeni[1], *Sanah Bucibo[1], Sandile Mthembu[1], *Sashen Moodley[1], Sashin Harilall[1], Senamile Makhari[1], Seneme Mchunu[1], Senzeni Mkhwanazi[1], Sibahle Gumbi[1], Siboniso Nene[1], Sibusiso Mhlongo[1], Sibusiso Mkhwanazi[1], Sibusiso Nsibande[1], Simphiwe Ntshangase[1], Siphephelo Dlamini[1], Sithembile Ngcobo[1], Siyabonga Nsibande[1], *Siyabonga Nxumalo[1], Sizwe Ndlela[1], Skhumbuzo Mthombeni[1], Smangaliso Zulu[1], Sphiwe Clement Mthembu[1], Sphiwe Ntuli[1], *Stephen Olivier[1], Talente Ntimbane[1], Thabile Zondi[1], *Thandeka Khoza[1], Thengokwakhe Nkosi[1], *Theresa Smit[1], Thokozani Bhengu[1], Thokozani Simelane[1], *Thumbi Ndung'u[1,5,7,8,9], *Tshwaraganang Modise[1], Tumi Madolo[1], Velile Vellem[14], Welcome Petros Mthembu[1], *Willem Hanekom[1], Xolani Mkhize[1], Zamashandu Mbatha[1], Zinhle Buthelezi[1], Zinhle Mthembu[1],*Zizile Sikhosana[1]

1 Africa Health Research Institute, KwaZulu-Natal, South Africa
2 Digital Engineering Faculty, Hasso Plattner Institute for Digital Engineering and University of Potsdam
3 Hasso Plattner Institute for Digital Health at Mount Sinai, Icahn School of Medicine at Mount Sinai, New York, NY, USA.
4 School of Nursing and Public Health, College of Health Sciences, University of KwaZulu-Natal, South Africa
5 Division of Infection and Immunity, University College London, London, UK
6 London School of Hygiene & Tropical Medicine, London, UK
7 HIV Pathogenesis Programme, The Doris Duke Medical Research Institute, University of KwaZulu-Natal, Durban, South Africa.
8 Ragon Institute of MGH, MIT and Harvard University, Cambridge, MA, USA.
9 Max Planck Institute for Infection Biology, Berlin, Germany.
10 School of Clinical Medicine, College of Health Sciences, University of KwaZulu-Natal, South Africa
11 Harvard Medical School, Boston, MA, USA.
12 Division of Infectious Diseases, Massachusetts General Hospital, Boston, MA, USA.
13 Division of Infectious Diseases, University of Alabama at Birmingham, AL, USA.
14 Aurum Innova (Pty) Ltd, Parktown, Johannesburg, South Africa
15 iMarketing Consultants, Windhoek West, Windhoek, Namibia
16 Perumal and Partners, Mayville, Durban, South Africa
17 Department of Pulmonology, Inkosi Albert Luthuli Central Hospital, Nelson R. Mandela School of Medicine, College of Health Sciences, University of KwaZulu-Natal, Durban, South Africa.
18 Traditional Medicine Laboratory, School of Nursing & Public Health, University of KwaZulu-Natal, South Africa
19 Jacpersad Inc, Overport, Durban, South Africa

**Supplemental Figures**

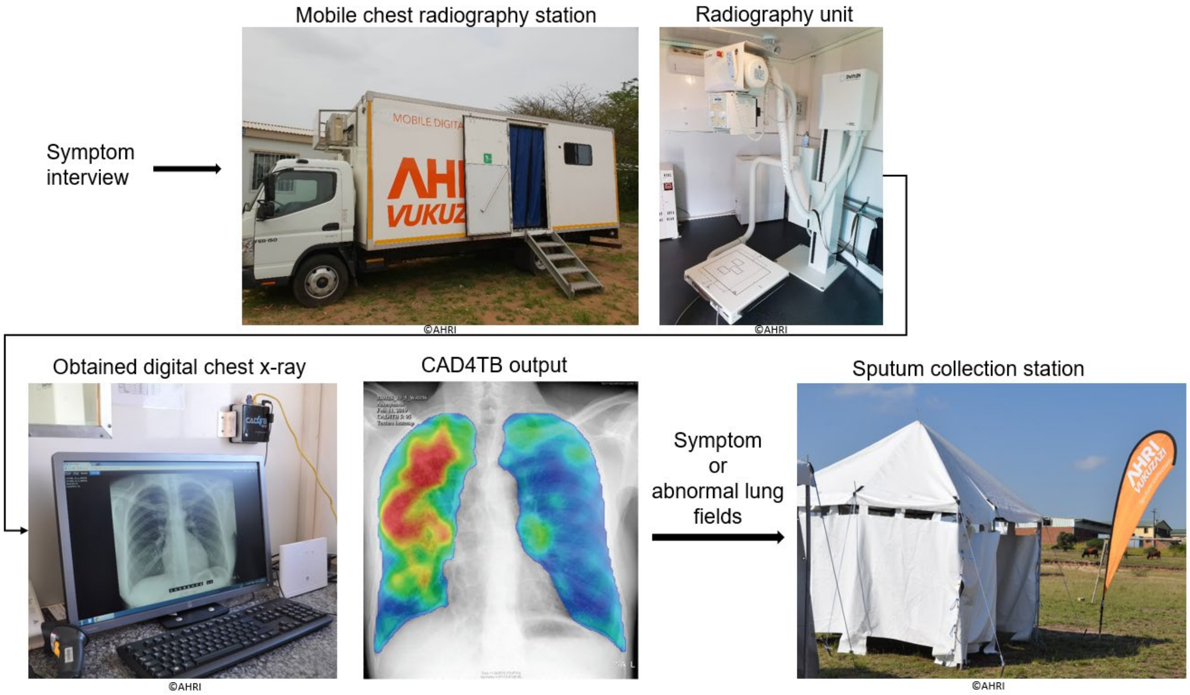


**Figure 1: Triaging for sputum assessment in Vukuzazi.** During the multi-morbidity health screening program Vukuzazi, 10,320 participants answered questions about current symptoms and underwent chest radiography in the mobile chest radiography station. The obtained digital chest x-rays were interpreted for present lung field abnormalities using the software CAD4TB. CAD4TB highlighted abnormal lung fields in the chest x-rays and presented those to the field radiographer. Participants were routed to sputum assessment station in the camp, if they reported TB-related symptoms (cough of any duration, fever, night sweat, or weight loss) or if CAD4TB detected lung field abnormalities. Photos ©AHRI.

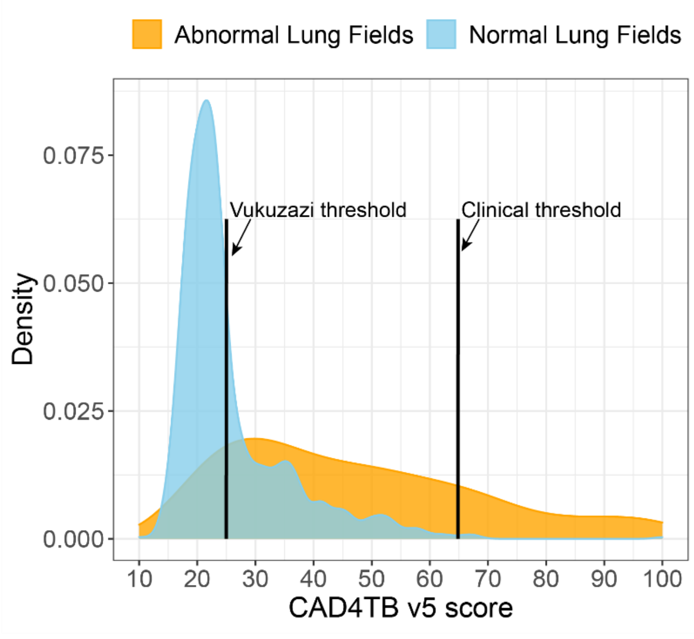


**Figure 2: Densities of CAD4TBv5 scores obtained during the pilot phase (n=1,090) stratified by the radiologist's classification of abnormal (n=198) and normal lung fields (n=892).** Triaging thresholds reported to be optimal in clinical thresholds are marked (65) next to the threshold chosen for this study (Vukuzazi) at 25.

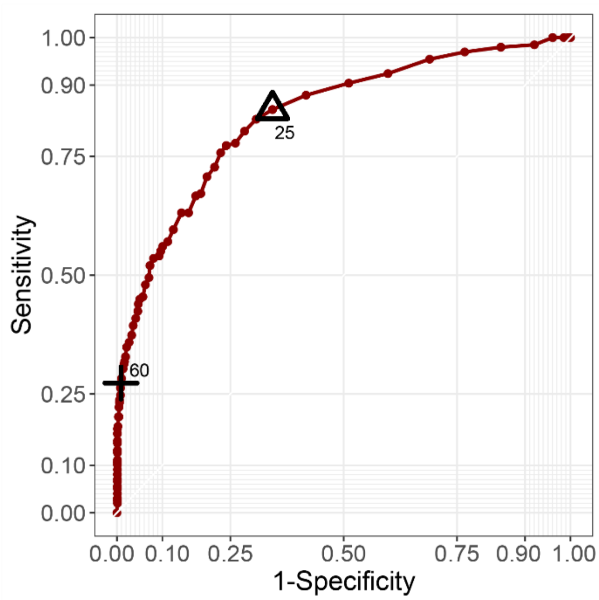


**Figure 3: Performance of CAD4TBv5 during the pilot-phase to detect CXRs classified as 'abnormal lung fields' by the radiologist.** CAD4TBv5 scores (n=1,090) were compared to the radiologist's classification of normal (n=892) and abnormal lung fields (n=198).

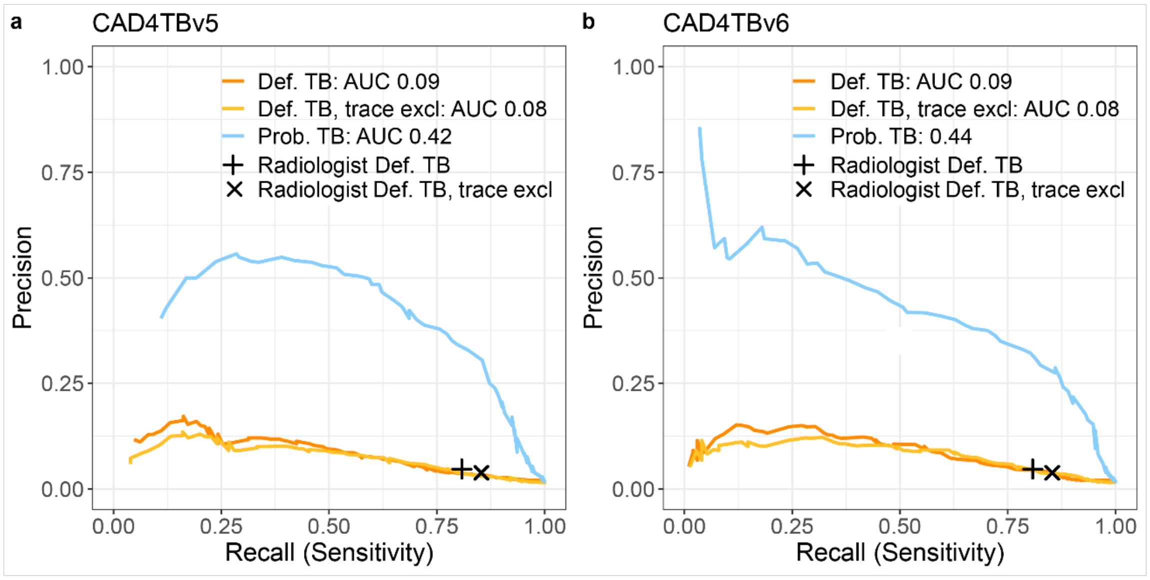


**Figure 4: Precision-Recall curve of CAD4TBv5 (a) and v6 (b) scores compared to diagnostic microbiological and/or radiological evidence.** Microbiological sputum test results were available for 4,976 participants. Radiological evidence was assessed among all participants who underwent chest radiography (n=9,914). Positive TB was defined as either definite TB with microbiological evidence definite TB excluding individuals that only had a XpertUltra trace result, or probable TB with radiological evidence of active TB but no microbiological evidence. The radiologist's precision and recall of detecting definite TB (+) and definite TB trace excluded is marked (x).

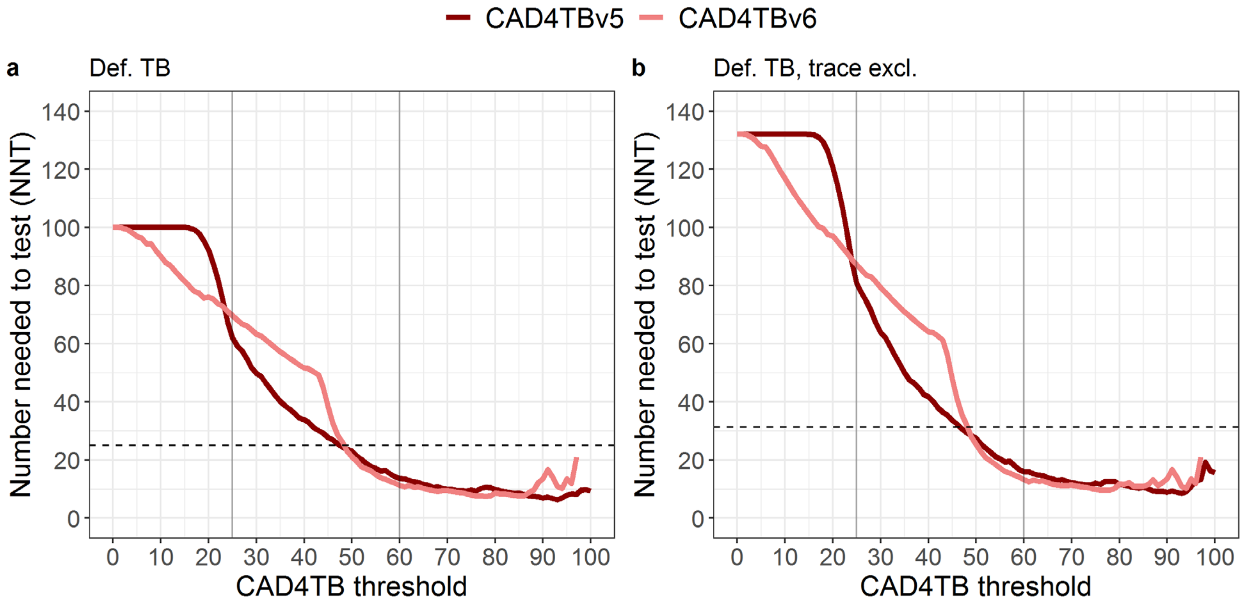


**Figure 5: Number needed to test (NNT) to find one participant with TB.** (a) The NNT to identify a participant with definite TB (n=99) or (b) definite TB trace excluded (n=75). NNT is the number of participants with a CAD4TB score equal or above each threshold divided by the number of identified definite TB at the respective threshold. The dashed line indicates the NNT of the radiologist (a: 25, b: 31).

**Supplemental Tables**

**Table 1: Performance of the radiologist and CAD4TBv5 and v6 to identify definite TB.** Performance among participants with microbiological sputum test results (n=4,976). Definite TB was defines as either positive XpertUltra or liquid culture test result (n=99). Performance is given as sensitivity, specificity, positive predictive value (PPV) and negative predictive value (NPV) in % with 95% confidence intervals (CI), number of participants need to test and missed definite TB cases. Further listed are the percentage of participants who required sputum testing due to lung field abnormality, deemed by the radiologist and CAD4TB and the number of needed tests (NNT) to find one participant with definite TB. Numbers of missed definite TB cases (all and asymptomatic) are listed as absolute numbers and relative to all definite TB cases (n=99).

| | **Sensitivity % (CI)** | | **Specificity % (CI)** | | **PPV % (CI)** | | **NPV % (CI)** | | **Participants requiring sputum testing (%)** | | **NNT** | | **#missed definite TB (%)** | | **#missed asymptomatic definite TB (%)** | |
|---|---|---|---|---|---|---|---|---|---|---|---|---|---|---|---|---|
| **Radiologist:** Any CXR abnormality | 80.8 (71.7-88.0) | | 66.9 (65.6-68.2) | | 4.7 (3.8-5.8) | | 99.4 (99.1-99.7) | | 2,002 (20.2) | | 25 | | 19 (19.2) | | 15 (15.2) | |
| CXR diag. active TB | 30.3 (21.5-40.4) | | 97.0 (96.5-97.4) | | 16.9 (11.7-23.3) | | 98.6 (98.2-98.9) | | 202 (2.0) | | 6.7 | | 69 (69.7) | | 55 (55.6) | |
| **CAD4TB** | **v5** | **v6** | **v5** | **v6** | **v5** | **v6** | **v5** | **v6** | **v5** | **v6** | **v5** | **v6** | **v5** | **v6** | **v5** | **v6** |
| 20 | 99.0 (94.5-100) | 92.9 (86-97.1) | 1.4 (1.1-1.7) | 11.7 (10.8-12.6) | 2.0 (1.6-2.4) | 2.1 (1.7-2.6) | 98.5 (92.1-100) | 98.8 (97.5-99.5) | 9,047 (91.3) | 6,990 (70.5) | 92 | 76 | 1 (1.0) | 7 (7.0) | 0 (0.0) | 5 (5.1) |
| 25 | 96.0 (90.0-98.9) | 90.9 (83.4-95.8) | 8.0 (7.3-8.8) | 16.9 (15.9-18) | 2.1 (1.7-2.5) | 2.2 (1.8-2.7) | 99 (97.4-99.7) | 98.9 (98-99.5) | 5,906 (59.6) | 6,276 (63.3) | 62 | 70 | 4 (4.0) | 9 (9.1) | 1 (1.0) | 6 (6.1) |
| 30 | 91.9 (84.7-96.4) | 89.9 (82.2-95) | 28.7 (27.5-30.0) | 22.4 (21.2-23.6) | 2.6 (2.1-3.1) | 2.3 (1.8-2.8) | 99.4 (98.9-99.8) | 99.1 (98.3-99.6) | 4,532 (45.7) | 5,635 (56.84) | 50 | 63 | 8 (8.1) | 10 (10.1) | 5 (5.1) | 7 (7.1) |

| | | | | | | | | | | | | | | | | |
|---|---|---|---|---|---|---|---|---|---|---|---|---|---|---|---|---|
| 35 | 86.9 (78.6-92.8) | 88.9 (81-94.3) | 45.6 (44.2-47) | 28 (26.7-29.3) | 3.1 (2.5-3.9) | 2.4 (2.0-3.0) | 99.4 (99-99.7) | 99.2 (98.6-99.6) | 3,459 (34.8) | 5,042 (50.9) | 40 | 57 | 13 (13.1) | 11 (11.1) | 10 (10.1) | 8 (8.1) |
| 40 | 79.8 (70.5-87.2) | 88.9 (81.0-94.3) | 57.4 (56-58.8) | 33.3 (31.9-34.6) | 3.7 (2.9-4.5) | 2.6 (2.1-3.2) | 99.3 (98.9-99.6) | 99.3 (98.8-99.7) | 2,677 (27.0) | 4,550 (45.9) | 34 | 52 | 20 (20.2) | 11 (11.1) | 14 (14.1) | 8 (8.1) |
| 41 | 77.8 (68.3-85.5) | 87.9 (79.8-93.6) | 59.7 (58.4-61.1) | 34.2 (32.8-35.5) | 3.8 (3.0-4.7) | 2.6 (2.1-3.2) | 99.3 (98.9-99.5) | 99.3 (98.8-99.6) | 2,533 (25.5) | 4,468 (45.1) | 33 | 51 | 22 (22.2) | 12 (12.1) | 16 (16.2) | 9 (9.1) |
| 42 | 77.8 (68.3-85.5) | 87.9 (79.8-93.6) | 61.7 (60.3-63.1) | 35.1 (33.7-36.4) | 4.0 (3.1-4.9) | 2.7 (2.1-3.3) | 99.3 (98.9-99.5) | 99.3 (98.8-99.6) | 2,397 (24.2) | 4,387 (44.3) | 31 | 50 | 22 (22.2) | 12 (12.1) | 16 (16.2) | 9 (9.1) |
| 43 | 75.8 (66.1-83.8) | 87.9 (79.8-93.6) | 63.6 (62.3-65) | 36.2 (34.8-37.5) | 4.1 (3.2-5.1) | 2.7 (2.1-3.3) | 99.2 (98.9-99.5) | 99.3 (98.8-99.6) | 2,264 (22.8) | 4,288 (43.3) | 30 | 49 | 24 (24.2) | 12 (12.1) | 17 (17.2) | 9 (9.1) |
| 44 | 74.7 (65.0-82.9) | 87.9 (79.8-93.6) | 65.3 (64-66.7) | 40.6 (39.2-42) | 4.2 (3.3-5.2) | 2.9 (2.3-3.6) | 99.2 (98.9-99.5) | 99.4 (99-99.7) | 2,167 (21.9) | 3,927 (39.6) | 29 | 45 | 25 (25.3) | 12 (12.1) | 18 (18.2) | 9 (9.1) |
| 45 | 74.7 (65.0-82.9) | 86.9 (78.6-92.8) | 67.2 (65.9-68.5) | 48.8 (47.4-50.3) | 4.4 (3.5-5.5) | 3.3 (2.7-4.1) | 99.2 (98.9-99.5) | 99.5 (99.1-99.7) | 2,051 (20.7) | 3,295 (33.2) | 28 | 38 | 25 (25.3) | 13 (13.1) | 18 (18.2) | 10 (10.1) |
| 46 | 72.7 (62.9-81.2) | 85.9 (77.4-92) | 68.9 (67.6-70.2) | 55.8 (54.4-57.2) | 4.5 (3.6-5.7) | 3.8 (3.0-4.7) | 99.2 (98.8-99.5) | 99.5 (99.1-99.7) | 1,943 (19.6) | 2,799 (28.2) | 27 | 33 | 27 (27.3) | 14 (14.1) | 20 (20.2) | 11 (11.1) |
| 47 | 72.7 (62.9-81.2) | 82.8 (73.9-89.7) | 70.5 (69.2-71.8) | 62.6 (61.2-64) | 4.8 (3.8-6.0) | 4.3 (3.4-5.3) | 99.2 (98.8-99.5) | 99.4 (99.1-99.7) | 1,842 (18.6) | 2,348 (23.7) | 26 | 29 | 27 (27.3) | 17 (17.2) | 20 (20.2) | 13 (13.1) |

| | | | | | | | | | | | | | | | | |
|---|---|---|---|---|---|---|---|---|---|---|---|---|---|---|---|---|
| 48 | 72.7 (62.9-81.2) | 76.8 (67.2-84.7) | 71.8 (70.5-73.1) | 68.0 (66.7-69.3) | 5.0 (3.9-6.2) | 4.6 (3.7-5.8) | 99.2 (98.8-99.5) | 99.3 (99-99.6) | 1,762 (17.8) | 1,997 (20.1) | 25 | 26 | 27 (27.3) | 23 (23.2) | 20 (20.2) | 18 (18.2) |
| 49 | 71.7 (61.8-80.3) | 74.7 (65-82.9) | 73.2 (72-74.5) | 72.6 (71.4-73.9) | 5.2 (4-6.5) | 5.3 (4.1-6.6) | 99.2 (98.8-99.5) | 99.3 (99-99.6) | 1,684 (17.0) | 1,701 (17.2) | 24 | 23 | 28 (28.3) | 25 (25.3) | 21 (21.2) | 19 (19.2) |
| 50 | 69.7 (59.6-78.5) | 70.7 (60.7-79.4) | 74.5 (73.3-75.8) | 76.4 (75.1-77.5) | 5.3 (4.1-6.6) | 5.7 (4.5-7.2) | 99.2 (98.8-99.4) | 99.2 (98.9-99.5) | 1,598 (16.1) | 1,474 (14.9) | 23 | 21 | 30 (30.3) | 29 (29.3) | 23 (23.2) | 22 (22.2) |
| 60 | 55.6 (45.2-65.5) | 55.6 (45.2-65.5) | 87.9 (87.0-88.8) | 89.9 (89.0-90.7) | 8.6 (6.5-11) | 10.1 (7.7-12.9) | 99.0 (98.6-99.3) | 99.0 (98.7-99.3) | 751 (7.6) | 623 (6.3) | 14 | 11 | 44 (44.4) | 44 (44.4) | 35 (35.4) | 34 (34.3) |
| 70 | 40.4 (30.7-50.7) | 36.4 (26.9-46.6) | 93.5 (92.8-94.2) | 94.6 (93.9-95.2) | 11.3 (8.2-15.0) | 12.0 (8.5-16.2) | 98.7 (98.4-99.0) | 98.7 (98.3-99.0) | 401 (4.0) | 342 (3.4) | 10 | 10 | 59 (59.6) | 63 (63.6) | 45 (45.5) | 49 (49.5) |
| 80 | 24.2 (16.2-33.9) | 19.2 (12.0-28.3) | 96.2 (95.6-96.7) | 97.4 (97.0-97.9) | 11.5 (7.5-16.6) | 13.2 (8.1-19.8) | 98.4 (98.0-98.8) | 98.3 (97.9-98.7) | 234 (2.4) | 165 (1.7) | 10 | 9 | 75 (75.8) | 80 (80.8) | 60 (60.6) | 64 (64.6) |
| 90 | 19.2 (12.0-28.3) | 4.0 (1.1-10) | 97.9 (97.5-98.3) | 99.1 (98.8-99.3) | 16.0 (9.9-23.8) | 8.3 (2.3-20.0) | 98.4 (98.0-98.7) | 98.1 (97.6-98.4) | 133 (1.3) | 54 (0.5) | 7 | 14 | 80 (80.8) | 95 (96.0) | 65 (65.7) | 75 (75.8) |

**Table 2: Performance of the radiologist and CAD4TBv5 and v6 to identify definite TB trace excluded.** Performance among participants with microbiological sputum test results, excluding participants who were only definite TB positive due to a XpertUltra trace result (remaining n=4,952). Performance is given as sensitivity, specificity, positive predictive value (PPV) and negative predictive value (NPV) in % with 95% confidence intervals (CI). Further listed are the percentage of participants who required sputum testing due to lung field abnormality, deemed by the radiologist and CAD4TB and the number of needed tests (NNT) to find one participant with definite TB, trace excluded. Numbers of missed definite TB cases (all and asymptomatic) are listed as absolute numbers and relative to all definite TB cases trace excluded (n=75).

| | **Sensitivity % (CI)** | | **Specificity % (CI)** | | **PPV % (CI)** | | **NPV % (CI)** | | **Participants requiring sputum testing (%)** | | **NNT** | | **#missed definite TB, trace excluded (%)** | | **#missed asymptomatic, trace excluded (%)** | |
|---|---|---|---|---|---|---|---|---|---|---|---|---|---|---|---|---|
| **Radiologist**: Any CXR abnormality | 85.3 (75.3-92.4) | | 66.9 (65.6-68.2) | | 3.8 (3.0-4.8) | | 99.7 (99.4-99.8) | | 2,002 (20.2) | | 31 | | 11 (14.7) | | 9 (12.0) | |
| CXR diag. active TB | 33.8 (23.0-46.0) | | 97.0 (96.5-97.4) | | 14.0 (9.2-20.2) | | 99.0 (98.7-99.3) | | 202 (2.0) | | 8 | | 50 (50.5) | | 41 (41.4) | |
| **CAD4TB score** | **v5** | **v6** | **v5** | **v6** | **v5** | **v6** | **v5** | **v6** | **v5** | **v6** | **v5** | **v6** | **v5** | **v6** | **v5** | **v6** |
| 20 | 100 (95.2-100) | 96 (88.8-99.2) | 1.4 (1.1-1.7) | 11.7 (10.8-12.6) | 1.5 (1.2-1.9) | 1.6 (1.3-2.1) | 100 (94.6-100) | 99.5 (98.5-99.9) | 9,047 (91.3) | 6,990 (70.5) | 121 | 97 | 0 (0.0) | 3 (4.0) | 0 (0.0) | 2 (2.7) |
| 25 | 97.3 (90.7-99.7) | 96.0 (88.8-99.2) | 8.0 (7.3-8.8) | 16.9 (15.9-18) | 1.6 (1.3-2) | 1.7 (1.4-2.2) | 99.5 (98.2-99.9) | 99.6 (99.1-99.9) | 5,906 (59.6) | 6,276 (63.3) | 81 | 87 | 2 (2.7) | 3 (4.0) | 1 (1.3) | 2 (2.7) |
| 30 | 94.7 (86.9-98.5) | 94.7 (86.9-98.5) | 28.7 (27.5-30.0) | 22.4 (21.2-23.6) | 2.0 (1.6-2.5) | 1.8 (1.4-2.3) | 99.7 (99.3-99.9) | 99.6 (99.1-99.9) | 4,532 (45.7) | 5,635 (56.8) | 64 | 79 | 4 (5.3) | 4 (5.3) | 3 (4.0) | 3 (4.0) |
| 35 | 92.0 (83.4-97.0) | 94.7 (86.9-98.5) | 45.6 (44.2-47.0) | 28.0 (26.7-29.3) | 2.5 (2-3.2) | 2.0 (1.6-2.5) | 99.7 (99.4-99.9) | 99.7 (99.2-99.9) | 3,459 (34.8) | 5,042 (50.9) | 50 | 71 | 6 (8.0) | 4 (5.3) | 5 (6.7) | 3 (4.0) |
| 40 | 85.3 (75.3-92.4) | 94.7 (86.9-98.5) | 57.4 (56-58.8) | 33.3 (31.9-34.6) | 3.0 (2.3-3.8) | 2.1 (1.7-2.7) | 99.6 (99.3-99.8) | 99.8 (99.4-99.9) | 2,677 (27.0) | 4,550 (45.9) | 42 | 64 | 11 (14.7) | 4 (5.3) | 8 (10.7) | 3 (4.0) |
| 45 | 81.3 (70.7-89.4) | 92.0 (83.4-97.0) | 67.2 (65.9-68.5) | 48.8 (47.4-50.3) | 3.7 (2.8-4.7) | 2.7 (2.1-3.4) | 99.6 (99.3-99.8) | 99.7 (99.5-99.9) | 2,051 (20.7) | 3,295 (33.2) | 34 | 48 | 14 (18.7) | 6 (8.0) | 11 (14.7) | 5 (6.7) |
| 50 | 77.3 (66.2-86.2) | 77.3 (66.2-86.2) | 74.5 (73.3-75.8) | 76.4 (75.1-77.5) | 4.5 (3.4-5.7) | 4.8 (3.7-6.1) | 99.5 (99.3-99.7) | 99.5 (99.3-99.7) | 1,598 (16.1) | 1,474 (14.9) | 23 | 21 | 17 (22.7) | 17 (22.7) | 14 (18.7) | 13 (17.3) |

| | | | | | | | | | | | | | | | | |
|---|---|---|---|---|---|---|---|---|---|---|---|---|---|---|---|---|
| 60 | 62.7 (50.7-73.6) | 62.7 (50.7-73.6) | 87.9 (87-88.8) | 89.9 (89-90.7) | 7.4 (5.5-9.7) | 8.7 (6.5-11.4) | 99.4 (99.1-99.6) | 99.4 (99.1-99.6) | 751 (7.6) | 623 (6.3) | 14 | 11 | 28 (37.3) | 28 (37.3) | 23 (30.7) | 22 (29.3) |
| 70 | 44.0 (32.5-55.9) | 40.0 (28.9-52.0) | 93.5 (92.8-94.2) | 94.6 (93.9-95.2) | 9.5 (6.6-13.1) | 10.2 (7.0-14.2) | 99.1 (98.8-99.3) | 99.0 (98.7-99.3) | 401 (4.0) | 342 (3.4) | 10 | 10 | 42 (56.0) | 45 (60.0) | 32 (42.7) | 35 (46.7) |
| 80 | 26.7 (17.1-38.1) | 18.7 (10.6-29.3) | 96.2 (95.6-96.7) | 97.4 (97-97.9) | 9.8 (6.1-14.7) | 10.1 (5.6-16.3) | 98.8 (98.5-99.1) | 98.7 (98.4-99) | 234 (2.4) | 165 (1.7) | 10 | 9 | 55 (73.3) | 61 (81.3) | 44 (58.7) | 49 (65.3) |
| 90 | 20.0 (11.6-30.8) | 5.3 (1.5-13.1) | 97.9 (97.5-98.3) | 99.1 (98.8-99.3) | 13.0 (7.5-20.6) | 8.3 (2.3-20.0) | 98.8 (98.4-99.1) | 98.6 (98.2-98.9) | 133 (1.3) | 54 (0.5) | 7 | 14 | 60 (80.0) | 71 (94.7) | 49 (65.3) | 56 (74.7) |

**Table S3: Performance of CAD4TBv5 to identify probable TB.** Performance among participants who underwent chest radiography (n=9,914). Probable TB was defined as radiological evidence indicated by the radiologist by 'CXR diagnostic of active TB' but no microbiological evidence (n=172). Performance is given as sensitivity, specificity, positive predictive value (PPV) and negative predictive value (NPV) in % with 95% confidence intervals (CI). The numbers of missed probable TB cases (all and asymptomatic) are listed as absolute numbers absolute and relative to all probable TB cases (n=172).

| **CAD4TB score** | **Sensitivity % (CI)** | | **Specificity % (CI)** | | **PPV % (CI)** | | **NPV % (CI)** | | **#missed probable TB (%)** | | **#missed asymptomatic probable TB (%)** | |
|---|---|---|---|---|---|---|---|---|---|---|---|---|
| | **v5** | **v6** | **v5** | **v6** | **v5** | **v6** | **v5** | **v6** | **v5** | **v6** | **v5** | **v6** |
| 20 | 100 (97.9-100) | 99.4 (96.8-100) | 8.9 (8.3-9.5) | 30.0 (29.1-30.9) | 1.9 (1.6-2.2) | 2.4 (2.1-2.8) | 100 (99.6-100) | 100 (99.8-100) | 0 (0.0) | 1 (0.6) | 0 (0.0) | 1 (0.6) |
| 25 | 99.4 (96.8-100) | 98.8 (95.9-99.9) | 41.1 (40.2-42.1) | 37.3 (36.4-38.3) | 2.9 (2.5-3.4) | 2.7 (2.3-3.1) | 100 (99.9-100) | 99.9 (99.8-100) | 1 (0.6) | 2 (1.2) | 1 (0.6) | 2 (1.2) |
| 30 | 97.7 (94.2-99.4) | 98.8 (95.9-99.9) | 55.2 (54.2-56.2) | 43.9 (42.9-44.9) | 3.7 (3.2-4.3) | 3 (2.6-3.5) | 99.9 (99.8-100) | 100 (99.8-100) | 4 (2.3) | 2 (1.2) | 4 (2.3) | 2 (1.2) |
| 35 | 97.1 (93.3-99) | 98.3 (95.0-99.6) | 66.2 (65.3-67.1) | 50.0 (49.0-51.0) | 4.8 (4.1-5.6) | 3.4 (2.9-3.9) | 99.9 (99.8-100) | 99.9 (99.8-100) | 5 (2.9) | 3 (1.7) | 5 (2.9) | 3 (1.7) |
| 40 | 95.9 (91.8-98.3) | 98.3 (95.0-99.6) | 74.2 (73.3-75.1) | 55.0 (54.0-56.0) | 6.2 (5.3-7.1) | 3.7 (3.2-4.3) | 99.9 (99.8-100) | 99.9 (99.8-100) | 7 (4.1) | 3 (1.7) | 6 (3.5) | 3 (1.7) |
| 45 | 95.3 (91.0-98.0) | 97.7 (94.2-99.4) | 80.6 (79.8-81.4) | 67.9 (67-68.8) | 8.0 (6.9-9.3) | 5.1 (4.4-5.9) | 99.9 (99.8-100) | 99.9 (99.8-100) | 8 (4.7) | 4 (1.7) | 7 (4.1) | 4 (2.3) |
| 50 | 93.6 (88.8-96.8) | 95.3 (91.0-98.0) | 85.2 (84.5-85.9) | 86.6 (85.9-87.2) | 10.1 (8.6-11.7) | 11.1 (9.6-12.8) | 99.9 (99.8-99.9) | 99.9 (99.8-100) | 11 (6.4) | 8 (4.7) | 10 (5.8) | 7 (4.1) |
| 60 | 90.1 (84.6-94.1) | 87.8 (81.9-92.3) | 93.9 (93.4-94.4) | 95.2 (94.7-95.6) | 20.6 (17.8-23.7) | 24.2 (20.9-27.8) | 99.8 (99.7-99.9) | 99.8 (99.7-99.9) | 17 (9.9) | 21 (12.2) | 14 (8.1) | 18 (10.5) |
| 70 | 79.7 (72.9-85.4) | 72.1 (64.8-78.7) | 97.3 (96.9-97.6) | 97.8 (97.4-98.0) | 34.2 (29.5-39) | 36.3 (31.2-41.6) | 99.6 (99.5-99.7) | 99.5 (99.3-99.6) | 35 (20.3) | 48 (27.9) | 27 (15.7) | 39 (22.7) |

| | | | | | | | | | | | | |
|---|---|---|---|---|---|---|---|---|---|---|---|---|
| 80 | 62.8 (55.1-70) | 44.8 (37.2-52.5) | 98.7 (98.5-98.9) | 99.1 (98.9-99.3) | 46.2 (39.6-52.8) | 46.7 (38.9-54.6) | 99.3 (99.2-99.5) | 99.0 (98.8-99.2) | 64 (37.2) | 95 (55.2) | 46 (26.7) | 68 (39.5) |
| 90 | 41.9 (34.4-49.6) | 18.6 (13.1-25.2) | 99.4 (99.2-99.5) | 99.8 (99.7-99.9) | 54.1 (45.3-62.8) | 59.3 (45-72.4) | 99.0 (98.8-99.2) | 98.6 (98.3-98.8) | 100 (58.1) | 140 (81.4) | 74 (43.0) | 104 (60.5) |

**Table 3: Performance of CAD4TBv5 to identify probable TB.** Probable TB was defined as radiological evidence indicated by the radiologist by 'CXR diagnostic of active TB' but no microbiological evidence. Performance is given as sensitivity, specificity, positive predictive value (PPV) and negative predictive value (NPV) in % with 95% confidence intervals (CI). The number of needed tests (NNT) is reported in absolute numbers and relative to all (n=9,914) participants. The number of all and asymptomatic missed definite TB cases is listed in absolute numbers and relative to all probable TB cases (n=172).

| **CAD4TB score** | **Sensitivity % (CI)** | | **Specificity % (CI)** | | **PPV % (CI)** | | **NPV % (CI)** | | **#missed probable TB (%)** | | **#missed asymptomatic probable TB (%)** | |
|---|---|---|---|---|---|---|---|---|---|---|---|---|
| | **v5** | **v6** | **v5** | **v6** | **v5** | **v6** | **v5** | **v6** | **v5** | **v6** | **v5** | **v6** |
| 20 | 100 (97.9-100) | 99.4 (96.8-100) | 8.9 (8.3-9.5) | 30.0 (29.1-30.9) | 1.9 (1.6-2.2) | 2.4 (2.1-2.8) | 100 (99.6-100) | 100 (99.8-100) | 0 (0.0) | 1 (0.6) | 0 (0.0) | 1 (0.6) |
| 25 | 99.4 (96.8-100) | 98.8 (95.9-99.9) | 41.1 (40.2-42.1) | 37.3 (36.4-38.3) | 2.9 (2.5-3.4) | 2.7 (2.3-3.1) | 100 (99.9-100) | 99.9 (99.8-100) | 1 (0.6) | 2 (1.2) | 1 (0.6) | 2 (1.2) |
| 30 | 97.7 (94.2-99.4) | 98.8 (95.9-99.9) | 55.2 (54.2-56.2) | 43.9 (42.9-44.9) | 3.7 (3.2-4.3) | 3 (2.6-3.5) | 99.9 (99.8-100) | 100 (99.8-100) | 4 (2.3) | 2 (1.2) | 4 (2.3) | 2 (1.2) |
| 35 | 97.1 (93.3-99) | 98.3 (95.0-99.6) | 66.2 (65.3-67.1) | 50.0 (49.0-51.0) | 4.8 (4.1-5.6) | 3.4 (2.9-3.9) | 99.9 (99.8-100) | 99.9 (99.8-100) | 5 (2.9) | 3 (1.7) | 5 (2.9) | 3 (1.7) |
| 40 | 95.9 (91.8-98.3) | 98.3 (95.0-99.6) | 74.2 (73.3-75.1) | 55.0 (54.0-56.0) | 6.2 (5.3-7.1) | 3.7 (3.2-4.3) | 99.9 (99.8-100) | 99.9 (99.8-100) | 7 (4.1) | 3 (1.7) | 6 (3.5) | 3 (1.7) |
| 45 | 95.3 (91.0-98.0) | 97.7 (94.2-99.4) | 80.6 (79.8-81.4) | 67.9 (67-68.8) | 8.0 (6.9-9.3) | 5.1 (4.4-5.9) | 99.9 (99.8-100) | 99.9 (99.8-100) | 8 (4.7) | 4 (1.7) | 7 (4.1) | 4 (2.3) |
| 50 | 93.6 (88.8-96.8) | 95.3 (91.0-98.0) | 85.2 (84.5-85.9) | 86.6 (85.9-87.2) | 10.1 (8.6-11.7) | 11.1 (9.6-12.8) | 99.9 (99.8-99.9) | 99.9 (99.8-100) | 11 (6.4) | 8 (4.7) | 10 (5.8) | 7 (4.1) |
| 60 | 90.1 (84.6-94.1) | 87.8 (81.9-92.3) | 93.9 (93.4-94.4) | 95.2 (94.7-95.6) | 20.6 (17.8-23.7) | 24.2 (20.9-27.8) | 99.8 (99.7-99.9) | 99.8 (99.7-99.9) | 17 (9.9) | 21 (12.2) | 14 (8.1) | 18 (10.5) |
| 70 | 79.7 (72.9-85.4) | 72.1 (64.8-78.7) | 97.3 (96.9-97.6) | 97.8 (97.4-98.0) | 34.2 (29.5-39) | 36.3 (31.2-41.6) | 99.6 (99.5-99.7) | 99.5 (99.3-99.6) | 35 (20.3) | 48 (27.9) | 27 (15.7) | 39 (22.7) |

| | | | | | | | | | | | |
|---|---|---|---|---|---|---|---|---|---|---|---|
| 80 | 62.8 (55.1-70) | 44.8 (37.2-52.5) | 98.7 (98.5-98.9) | 99.1 (98.9-99.3) | 46.2 (39.6-52.8) | 46.7 (38.9-54.6) | 99.3 (99.2-99.5) | 99.0 (98.8-99.2) | 64 (37.2) | 95 (55.2) | 46 (26.7) | 68 (39.5) |
| 90 | 41.9 (34.4-49.6) | 18.6 (13.1-25.2) | 99.4 (99.2-99.5) | 99.8 (99.7-99.9) | 54.1 (45.3-62.8) | 59.3 (45-72.4) | 99.0 (98.8-99.2) | 98.6 (98.3-98.8) | 100 (58.1) | 140 (81.4) | 74 (43.0) | 104 (60.5) |

**Table 4: Area under the receiver operating curves (AUC) from CAD4TBv5 and v6 compared to diagnostic microbiological and/or radiological evidence.** Positive TB was defined as either definite TB with microbiological evidence, definite TB excluding samples that only had a XpertUltra trace result, or probable TB with radiological signs of TB but no microbiological evidence.

| | **Definite TB** | **Definite TB trace excl.** | **Probable TB** |
|---|---|---|---|
| v5 AUC (CI) | 0.78 (0.73-0.83) | 0.82 (0.77-0.87) | 0.96 (0.95-0.98) |
| v5 AUC (CI) HIV-negative | 0.75 (0.68-0.83) | 0.81 (0.74-0.89) | 0.96 (0.93-0.98) |
| v5 AUC (CI) HIV-positive | 0.80 (0.72-0.87) | 0.82 (0.74-0.89) | 0.97 (0.96-0.99) |
| v6 AUC (CI) | 0.79 (0.73-0.84) | 0.84 (0.79-0.89) | 0.96 (0.95-0.98) |
| v6 AUC (CI) HIV-negative | 0.76 (0.68-0.84) | 0.83 (0.76-0.91) | 0.95 (0.93-0.98) |
| v6 AUC (CI) HIV-positive | 0.81 (0.74-0.88) | 0.82 (0.76-0.89) | 0.97 (0.97-0.98) |

**Table 5: P-values of comparing the diagnostic performance of CAD4TB to gold standards measured in area under the curve (AUC) between diagnostic groups.**

| | CAD4TBv5 | CAD4TBv6 |
|---|---|---|
| Definite TB vs. Definite TB, trace excluded | 0.28 | 0.18 |
| Definite TB vs. Probable TB | $5.25 \times 10^{-11}$ | $9.05 \times 10^{-10}$ |
| Definite TB, trace excluded vs. Probable TB | $4.51 \times 10^{-7}$ | $2.5 \times 10^{-6}$ |

**Table 6: P-values of comparing the diagnostic performance of CAD4TB to gold standards measured in area under the curve (AUC) between HIV-positive and negative individuals.** Gold standards were defined as either definite TB with microbiological evidence (M+), definite TB excluding M+ samples that only had a XpertUltra trace result, or probable TB with no microbiological evidence but radiological signs of TB (M-/0 R+). CAD4TB scores from version 5 (v5) and 6 (v6) were compared against these gold standards.

| **Gold standard** | **CAD4TBv5** | **CAD4TBv6** |
|---|---|---|
| Def. TB | 0.42 | 0.32 |
| Def. TB trace excl. | 0.96 | 0.85 |
| Prob. TB | 0.30 | 0.16 |
